# Views and experiences on the use of voice assistants by family and professionals supporting people with cognitive impairments

**DOI:** 10.3389/frdem.2022.1049464

**Published:** 2022-11-02

**Authors:** Ana-Maria Salai, Alexandra Kirton, Glenda Cook, Lars Erik Holmquist

**Affiliations:** ^1^Health and Life Sciences Faculty, Nursing, Midwifery & Health Department, Northumbria University, Newcastle upon Tyne, United Kingdom; ^2^School of Design, Northumbria University, Newcastle upon Tyne, United Kingdom

**Keywords:** dementia, family caregiver, voice assistants, smart home, dementia care, field study, older adults

## Abstract

The use of voice assistants (e.g., Amazon Alexa, Google Home) is being widely advocated as part of supporting people living with dementia at home. The development of this technology is largely driven by industry, and there is little research to determine how family carers and professionals use voice assistants with people with dementia. This paper presents the findings from further analysis of data from two studies: Study 1—a qualitative study that aimed to explore the views and expectations of family carers and professionals who use voice assistants to support people with a cognitive impairment at home, and Study 2—a qualitative enquiry aiming to identify the views and barriers on using voice assistants by family carers of people with dementia and professionals, together with a pilot case study evaluating a prototype that addresses barriers identified during the enquiry, entitled IntraVox. Based on processing of smart home sensor data, IntraVox uses a personalised human voice to send prompts and reminders to end-users to conduct daily life activities and to activate smart home processes using voice assistants. The results of the qualitative studies indicate that family carers and professionals use voice assistants in their caring role for home automation, skills maintenance and development, prompts and reminders, behaviour and environment monitoring, and for leisure and social interaction support. The findings also show that family carers and professionals have specific challenges that need to be overcome for them to realise the benefits that may be gained through the use of voice assistants within technology enabled care. The pilot case study also provided a useful demonstration that interoperability can be achieved to enable exchanges between IntraVox and voice assistants, with the aim of providing customised and personalised technological solutions that address some of the barriers that people with dementia and their carers face in the use of this technology.

## Introduction

Dementia is currently one of the major causes of disability and dependency amongst older adults that affects an individual's ability to interact with others and maintain a sense of self and identity (World Health Organization, 2022a). People with dementia often experience difficulties in performing daily activities such as cooking, washing, maintaining personal hygiene, and using appliances and devices (Rosenberg and Nygard, 2011; Rosenberg et al., 2012; Salai et al., 2021). In other words, dementia affects all aspects of an individual's life (World Health Organization, 2022b). The impact on caregivers and supporters is also well-recognised (World Health Organization, 2022c). It is no wonder that the technology industry has responded with the development of a broad range of technologies to support individuals as a distinct component of dementia care.

The technology enabled care revolution that is taking place across the globe has the potential to transform how people are supported in their homes and communities, and how care and health services are delivered (Honeyman et al., 2021). It is generally recognised that the majority of older people want to live in their own home and to age-in-place. However, conditions such as dementia make this increasingly difficult when symptoms increase and the behaviour changes as the condition advances (Satariano et al., 2014; Soilemezi et al., 2019). Across the globe the majority of people living with dementia are supported at home by family caregivers (Prince et al., 2015), and in some situations these caregivers draw on a range of digital resources to support them in their caring role.

In a recent two-round delphi study that explored which technologies would be most prevalent in dementia care in the next 5 years and assessed potential benefits and risks for older people with mild dementia, Berridge et al. (2021) concluded that smart home systems to control environmental settings and appliances would be the most prevalent technology. Indeed, amongst the digital products and services that are now available, there is a significant increase in the technology that utilises the Internet of Things (IoT), i.e., physical objects that connect and exchange data with each other over the Internet. This use of everyday objects as connected devices (named “*smart devices*”) can provide enhanced functionalities and benefits to householders. However, a smart home equipped with smart devices (sensors, smart light bulbs, smart TVs, smart kettles, smart blinds, etc.) requires a smart home hub that connects all the devices. Voice assistants, such as Amazon's Alexa and Google Home, are the most used smart hubs available on the market and have been embraced by consumers across the globe (Juniper Research, 2017). These devices can bring a lot of benefits to consumers and, in the context of older people's care, are being promoted as technologies that can provide home support (Salai et al., 2021). For example, the voice assistants can help with controlling lighting, sending reminders to users to conduct several tasks, offer video calling functionalities, or provide cooking instructions. Hence, voice assistants are proving to be a disruptive technology for businesses as well as health and care services due to the shift in the way that humans interact with technology leading to changes in business operating systems and changes in the care experience (Brill et al., 2019). It is increasingly recognised that much of this transformation is driven by industry, the danger being that products and services may not be usable, appropriate, and acceptable to the intended end-user (Kadylak and Shelia, 2020).

No large-scale trials or project evaluations exist within the IoT literature (Brill et al., 2019), though there are studies that conclude that smart technology functions could potentially be used for health monitoring (Maguire et al., 2021). This paper presents the findings from further analysis of two studies: *Study 1*—a qualitative study that aimed to explore the views and expectations of family carers and professionals who use voice assistants to support people at home who were living with a cognitive impairment, and *Study 2*—a two stage study including a qualitative enquiry aiming to identify the purposes of using voice assistants by family carers of people with dementia and professionals in their caring role and the barriers they face when interacting with such devices, together with a pilot case study evaluating a smart technological prototype that addresses barriers identified during the enquiry, entitled *IntraVox* (Salai et al., 2021).

## Methods and materials

In this section, we describe the methods that we applied whilst conducting the two studies mentioned above.

### Ethical considerations

Assistive technologies, particularly when used to support those with dementia, bring to light a multitude of ethical debates (Martin et al., 2010). Due to their ubiquitous nature—i.e., voice assistants are always listening—these ethical debates must be explored and should focus on the issue of informed consent and assent, and the protection of privacy and personal data from unconsented surveillance (Ienca et al., 2018). There is extensive ethical debate surrounding the use of monitoring technologies, such as voice assistants, in the care and support of people with dementia at home. This mainly refers to the balance between the ethical principles of autonomy, independence and privacy vs. beneficence or safety (Hall et al., 2019). Many studies report that both professionals and carers believe that monitoring technologies may protect people from harm while maintaining their autonomy (Mulvenna et al., 2017; Hall et al., 2019). Caregivers as well as those with dementia state that as the disease progresses, the need for health and safety monitoring increases (Dröes et al., 2006). Additionally, people with dementia report that they would prefer to “age-in-place” (Bharucha et al., 2009). Therefore, it can be reasonably deduced that, in general, people with dementia may consider use of this technology, at the expense of monitoring, if this enables them to remain in their own home and out of institutional care. In reality, carers are using monitoring technologies, such as voice assistants that are readily available on the open market, for care and support at home with some significant caveats around privacy and with limited scrutiny (Mulvenna et al., 2017). As UK law states, anything done on behalf of an individual lacking capacity must be the least restrictive form of intervention, and it could be argued that the use of monitoring technologies, or voice assistants, offers a less restrictive affront to a person's rights and freedom than movement from their own home (Mulvenna et al., 2017). It is therefore timely to explore how voice assistants are being used by carers to develop the evidence base and offer understanding of this approach to care in order to facilitate ethical practise.

### Study 1

#### Aim and approach

This study aimed to explore the views, experiences and expectations of family carers and professionals regarding the smart devices they use to support people living with a cognitive impairment in their own home. This study adopted a phenomenological, interpretivist framework to explore informal carers' and professionals' perspectives of engaging with smart devices in their caring roles. Phenomenology describes the lived experience of a particular phenomenon (Streubert and Carpenter, 2011), whereas interpretivism is a methodological approach which involves the researcher attempting to understand the meanings that humans attach to their experiences (Schwandt, 1994). Hence this methodological framework facilitated insight into the participant's experiences of a novel use of voice assistants as part of an assistive technology offer from local authority services.

#### Sample

A purposive sample was recruited to the primary study. The study was discussed with local authority social work and occupational therapists who were using smart devices as part of a care package, and those who were considering use of such technology, to determine interest in the study. Use of voice assistants in a package of care is relatively new in the UK therefore the target sample population was small. Their clients, family carers, were also invited to participate. Eleven family carers and 19 health and care professionals agreed to take part in the primary study, and of those, only two family carers and eight professionals had previous experience of voice assistants. The data relating to only those having experience of voice assistants in their caring role was extracted for the secondary analysis that is presented in this paper. These were family carers (F = 1, 52 years; M = 1, 60 years) who were caring for a man, 65 years old with frontal lobe, Alzheimer's and vascular dementia; and a carer of a woman, 82 years old with unspecified dementia. Of the professionals, there were five occupational therapists, one social worker, one assistant social worker, and one social worker/occupational therapist (F = 7; M = 1; age range 20–50 years old).

#### Data collection

The carers were invited to take part in a sequence of three semi-structured in-depth interviews over 3 months. This approach provided opportunity for in-depth discussion of their experiences over time, thus enhancing the richness of the data. The initial interview was to inquire into background experiences such as their use of smart technology. The following interviews were to explore ongoing use of technology and reflect on previous discussions. One of the participants completed three interviews, whereas only one completed two interviews because the person that they were caring for entered institutional care and decided to withdraw from the study.

The professionals were invited to take part in one of two focus group interviews. They were asked about their experiences of using smart technology to support carers of those with a cognitive impairment. They were asked about their views and use-cases for this technology, and barriers to acceptance of and use of this type of technology.

### Study 2

#### Aim and approach

The aims of this study were to (1) explore the views and experiences of professionals and family carers of people with dementia regarding the purposes for their use of voice assistants in their caring role; (2) identify barriers to use of voice assistants by people with dementia, professionals and family carers; (3) develop a technological intervention to address identified barriers and test the prototype through a pilot case study.

The study had two stages: *Stage 1*—a qualitative enquiry focusing on the first two aims presented above, and *Stage 2*—the development and evaluation of a prototype addressing identified barriers in Stage 1, entitled *IntraVox* (Salai et al., 2021). Based on processing of smart home sensor data, IntraVox uses a personalised human voice to send prompts and reminders to end-users to conduct daily life activities, and to activate smart home processes using voice assistants.

#### Sample

In *Stage 1*, we established a Community of Practise (CoP members) comprised of 24 members—two people with dementia (1 = F, 85 years; 1 = M, 94 years; both unspecified types of dementia as reported by their family carers), three family carers (live-in daughter of the female participant; live-in daughter and son-in-law of the male participant), four individuals from carer organisations, five Local Authority adult social care professionals, two health integration leads and commissioners, six National Health Service (NHS) older people's service managers, two NHS technology managers focusing on providing digital health care solutions to improve patient care.

*Stage 2* included one person with severe dementia and their family carer. These individuals were identified through discussion with a Local Authority provider that was aware that, although the client was using telecare and smart products, problems persisted. The client was a woman with advanced dementia, who lived alone at home (referred to as “*the mother*”). Her son provided care and stayed with her during evenings and mornings. Several carers (city council employees) visited the mother twice daily to prepare meals and provide medication prompts. The son gave his informed consent to take part in the study and he also acted as a consultee for the involvement of his mother who lacked the capacity to consent (Slaughter et al., 2007). This study occurred during a wave of COVID-19, therefore there was a requirement for social distancing and only those providing direct care were in contact with the client. As this was the first deployment of a novel technology in these circumstances the service provider supported the use of the technology, but the use was restricted to one client and their family carer. This is clearly a limitation that should be recognised, and care should be taken in interpreting the findings and guarding against transferring to other situations.

#### Data collection

In *Stage 1*, seven group interviews were conducted. We firstly conducted three group interviews with a mix of health, social care, technologist professionals, and carer organisation CoP members, followed by two group interviews comprised of the people with dementia and their family carers. Participants were asked about their personal experience and views of smart technology (e.g., purposes, barriers), with a focus on voice assistants. As this study was conducted at a time when there was a requirement for social distancing due to the global COVID-19 pandemic, all interviews were undertaken through the Microsoft Teams on-line platform (Saberi, 2020). Following analysis of this data, a technological solution, IntraVox (Salai et al., 2021), was developed to address barriers to use of voice assistants. In keeping with co-production methods a further two on-line interviews, were conducted with nine CoP members to explore their views of the initial IntraVox prototype. Design changes were subsequently made, such as type of voice used to activate the voice assistant.

*Stage 2* had two phases of data collection. In *Phase 1*, the pre-deployment of IntraVox, a semi-structured interview was carried out with the son to determine his familiarity with smart home devices, key issues in his caring role and how IntraVox could provide an intervention. Through this interview, it was identified that IntraVox should provide a prompt to alter the mothers behaviour when entering a food storage area. Following this initial interview, IntraVox was installed in the mother's home in compliance with COVID-19 pandemic procedures. The case study was then conducted for 7 days. In *Phase 2*, a post-study semi-structured interview was conducted to explore the son's views and experiences of IntraVox, and his views of the impact that IntraVox had on his mother's situation.

### Cross data analysis

Data from Study 1 and Stage 1 of Study 2 was transcribed verbatim and anonymised. Following Miles and Huberman (1994), a combination of systematic coding and open coding was employed during data analysis. First, the data from both studies was coded separately by two members of the team. Following this, the codes were compared to identify similarities and differences in the data sets. This led to refinement and development of the codes. This process confirmed that the participants from the different studies discussed the same issues, albeit in different contexts. This led to the development of two themes that were categorised by expansive master headings. These were Care practises augmented through voice assistants, and the findings derived from this process underpinned the development of IntraVox. The findings were not discussed with all of the participants due to lack of contact with them when this activity was being undertaken and restrictions presented by the pandemic, with only a limited number of CoP members contributing to the on-line discussions about IntraVox. These issues should be taken into consideration when interpretating the findings. Data collected during the pilot case study (Stage 2 of Study 2) was analysed using elements of thematic analysis by 2 members of the team to enhance rigour in the analytic process (Hsieh and Shannon, 2005).

## Results

### Care practises augmented through voice assistants

Across the studies the participants spoke of the various ways that they used voice assistants in their caring role:

*Because she'd forgotten how to heat the place we just kept an eye on it with the sensors and as I say in the next stage I would get fully in control of the heating*. (family carer)

*He used to get up and pee all over the floor. I didn't know. I would literally have to walk in it or smell it before I knew it had happened. So the sensor tells me that he's up out of bed via Alexa in the living room. Yeah. Alexa will say “Ben is getting up out of bed.”* (family carer)

Family carers spoke of the behaviours and actions of the person that they were caring for and how the voice assistant alerted them to situations that required attention or helped with automation of the home environment. Both family carers and professionals described how they used technologies for a range of purposes that could be categorised as:

- Home Automation—automating the house based on sensors and use of appliance data.
- Skills Maintenance and Development—supporting individuals to maintain and develop skills such as personal care, and housekeeping.
- Prompts and Reminders—providing alerts to support individuals to complete routine tasks or activities such as making a telephone call or attending an appointment.
- Behaviour and Environment Monitoring—providing family carers, and health and care professionals, access to personal activity and household data.
- Leisure and social interaction—keeping in touch with others and engaging in interests.

Participants also spoke of the complementarity of the voice assistant to their caring role. For example, the alerts Alexa can provide to family carers can enable them to undertake tasks in the home with personal reassurance and a sense of security knowing that the person they care for is safe in another area of the house. Other participants highlighted that voice assistants did not replace the human care that they provided:

*Now I've got that reassurance that Alexa tells me when the doors open. Erm I've got that reassurance that if I'm doing something in the kitchen and he does open the door I get notified whereas before I didn't have that. I could get straight to the front door and see what direction he's gone. I would then bring him back*. (family carer)

Similarly, those providing care remotely were able to balance safety and autonomy. Another family carer was increasingly concerned about the wellbeing of their mother, who wanted to continue to live in her own home, yet she had problems in self-care. The use of the Alexa Show was provided by a professional carer as an additional way of enabling the family carer to prompt the mother to take the prescribed medication. The family care suggested that the technology was “*easing the situation*,” and the situation was described by this professional carer:

*I've just had a case; it was a lady who required medication prompts. We'd provided her with an Alexa Show because the family were having to phone her up every day to remind her to take her medication. They were turning up and she still hadn't taken her medication even though she'd said that she had. So the Alexa Show means that the family can actually drop in on her (virtually) and actually watch her take her tablets*. (professional)

Providing care can be demanding and stressful which can impact negatively on a family carer's emotional wellbeing. Both family carers and professionals spoke of the way technology offered reassurance and practical support with everyday activities. Family carers were able to “*get on with life*.” The technology was also perceived as having the potential to be life enhancing for the person living with dementia:

*She's been playing music, she said she'd felt like she'd actually been out all morning because she'd been listening to the music over Alexa, so it was a really positive experience*. (professional)

It should be noted the ethical nuance that these insights offer. The feedback provides an articulation of the balance that must be struck between an ethical and moral approach when the right protocols are in place to protect privacy while maximising independence and safety.

### Barriers to adopting and using voice assistants

Whilst participants highlighted their perceptions and experiences of the utility of voice assistants, they were also very clear that there were obstacles to the use of such devices and their applications. Connectivity and robust access to Wi-Fi were common problems:

*All of these technologies run off Wi-Fi. You have all these ideas and go through the process of referring to the assistive technology team and then it's like no, the Wi-Fi signal isn't good enough. So, that was frustrating*. (professional)

Cost is another prohibiting factor:

*I was worried about how much it would cost*. (family carer)

*Thinking that the cost of living rises. Broadband is like £25 a month. Some people can't afford that unfortunately, so that's a stumbling block*. (professional)

Whilst some were challenged by digital poverty, others were concerned about digital competence. The professionals recognised that competence and confidence to use voice assistants could be a barrier to use, therefore training and support for family carers was deemed essential. However, a high level of digital competence could be undermined if the technology required use of multiple applications, non-friendly user interface, difficulty in navigation, or lack of specificity in determining end-user behaviour. These design issues were considered “*frustrating*” and adding burden to the caring role:

*I keep getting complaints. It's usually people with dementia, where they're up and down, so the sensors are setting off alarms constantly. I had one woman who, I think she had one hundred and something alarms in a month*. (professional)

In this situation there was a major impact on the family carer. In many other discussions there was as much concern about the reaction of the person living with dementia to the technology in their home:

*The only problem is, she keeps unplugging the Alexa because it's that fire risk. She's at that age where she's used to unplugging everything. They've* [the family] *found that putting little notes up next to the plug sockets, “do not unplug,” appears to be working at the moment*. (professional)

*I think the main problem I have come across is people just not recognising something with it being new. They say “this isn't mine, can you take it away?”; “I don't know what this is.” They just unplug it, wrap the wire up, and say “take it away, I don't know what it is. I don't understand it.”* (professional)

The lack of familiarity with devices and understanding how they work presented difficulty with use. Others described how those with memory problems struggle with recalling keywords and stating prompts such as “*Alexa/Hey Google, turn on the living room lights”* to activate the voice assistant. Family carers also suggested that Alexa could be difficult to hear, and the information provided by the assistant could be complicated. Some stated that Google Home and Alexa were “*very fast talkers and need to slow down*.” They argued that Alexa offers too much information that is “*difficult to retain in one go*.” They also found the instructions given by Alexa to be somewhat complicated. For example, they found it hard to remember the number of meal choices offered by Alexa. These views of voice assistants focus on the challenges that these carers had experienced when instructing and listening to a voice assistant. The other major area of concern was their observation of the person with dementia experiencing increased confusion and anxiety when hearing the virtual assistant in their home when others were not physically present:

*Hearing voices that they don't recognise and searching for that person*. (family carer)

### Technological solution to enable people with dementia and their carers to use voice assistants: IntraVox

**Figure 1:**
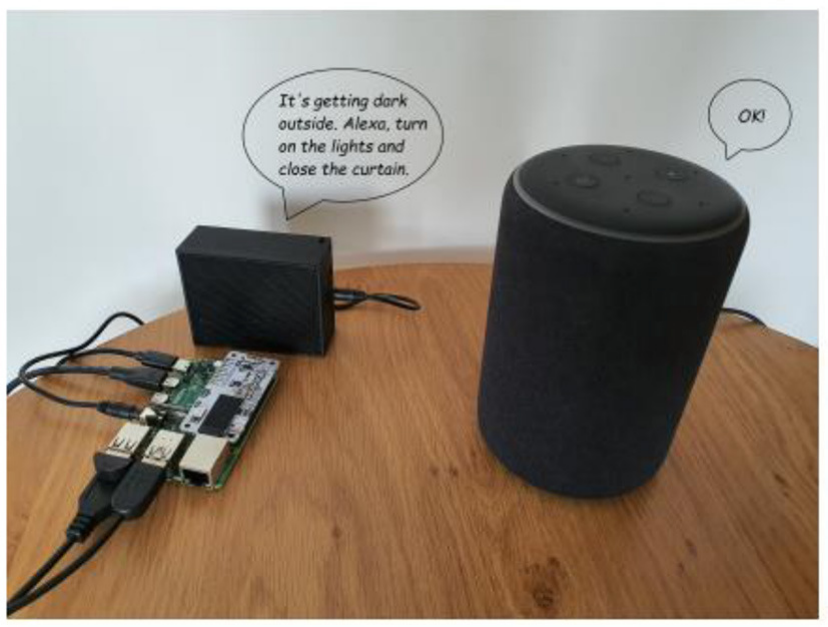
IntraVox is composed of a Raspberry Pi 4B, sensors, speaker, and an Amazon Alexa device. Reproduced with permission (Salai et al., 2021).

The previous findings suggest that virtual assistants can augment care and realise benefits for carers, yet there are barriers to use and limitations to this technology in a care context. To overcome activation problems and enhance the functionality of voice assistants to address the five technology support domains presented above, motion sensors that detect movement, and ambient sensors such as temperature, sound, and light were researched. These sensors were connected to a Raspberry Pi computer (Figure 1), a widely used single-board computer for home automation that allows easy access to the sensor data collected.

Software was written in the Python programming language to analyse the data collected from the sensors and send a message to control the voice assistant, or directly to the user. The message sent from the Raspberry Pi could be sent (1) silently using a third-party website that creates a virtual/silent Amazon Echo device (referred to as the *Silent System*), or (2) audibly by the Raspberry Pi using a synthesised voice created by a text-to-speech library (referred to as the *Synthesised Voice System*). As an example, based on the light sensor data that was collected, the Raspberry Pi can verbalise different types of prompts:

*Prompt 1*—a prompt to Alexa to trigger home processes: “*It is getting dark outside. Alexa, turn on the lights*.” or double commands “*Alexa, turn on the lights and close the curtains*.” These types of messages ensure that the person living in the house is informed that a home automation process is occurring.

*Prompt 2*—a prompt to suggest actions to the home occupant: “*It is getting dark outside. Maybe you should turn on the lights and close the curtains*.” This type of message promotes independent living through suggesting to the home occupant decisions to take action to change their environment.

The participants (see Section Materials and methods, Study 2, Stage 1) provided positive feedback with respect to the potential of this prototype to overcome some problems they experienced when using voice assistants, and they provided suggestions for improvement. They indicated that changes in the home simply happening without any notification could be “*confusing*” and “*frightening*” (in reference to the Silent System). Additionally, the voice used by the Synthesised Voice System could be perceived as “*unpleasant*” and “*frustrating*.” These revelations led to the development of *IntraVox*, a human voice-based interaction system. Similar to the Silent and Synthesised Voice Systems, IntraVox indicates what is going to happen in the house or provides prompts for actions through messages delivered *via* a human voice that is familiar to the end-user, such as a partner or carer. They also suggested that this approach can improve the usability of voice assistants by removing the necessity for the end-user to remember and pronounce specific commands. The personalised human voice of IntraVox, in particular, received positive feedback:

*It is helpful to program Alexa and Google Home to sound like a family member. This helps in personalising the support provided to the customer*. (CoP member)

*The familiar voice that IntraVox uses is good and the messages are delivered at a good pace*. (family carer)

#### Case study

Prior to the pilot case study, the son had installed multiple devices in his mother's house, such as smart cameras to monitor the paid carers visit times, USB-controlled gas, smart switches, and Amazon Alexa for controlling the lights and to support the mother to listen to the radio. He indicated that the technology had been installed in order to support his mother to stay in her own home, which had been her long-term aspiration. The devices were constantly active. However, the mother was unable to interact with any of the devices because of her advanced dementia. He described his mother's interaction with Alexa:

[Mum] *tries to have a conversation with Alexa, but the device doesn't answer. She gets bored after a while because Alexa doesn't answer. She calls Alexa “Thingy,” and when she hears an answer she asks me “What did Thingy say?”* (son—family carer)

He mentioned that the syntax required to enable Alexa limited his mother's interaction with the device. In particular, she was unable to learn the syntax required to turn on the television:

*It would be amazing if she would learn how to turn on the TV*. (son—family carer)

His mother's cognitive abilities had gradually deteriorated, and she needed support with various aspects of her daily life. One problem, that also impacted on his quality of life, was her frequent use of a food storage area as her bathroom. This was increasingly having a negative impact on their interaction, when much of their time he visited his mother was given to the task as cleaning her house, rather than engaging in personally meaningful interaction. When asked about the type of intervention he considered most appropriate he suggested that IntraVox could be useful in “*reminding her that's not the bathroom and she should go upstairs to use the toilet.”* Different audio prompts were considered (see Table 1) to play when the motion sensor, that was installed in the food storage area, was activated.

**Table 1:** Type of prompts and home automation activated by IntraVox.

**Type of prompt**	**Type of sensors and smart devices**	**Type of messages**	**SMART home process**
Prompt 1	Motion sensor Smart light bars	“*Alexa, turn on the guiding lights*.”	A light bar would turn on showing the way out of the storage room.
	Motion sensor Smart light bulbs	“*Alexa, turn on the toilet lights*.” “*Mary, the lights are on in the toilet*. *Please go there*.”	The lights in the bathroom would turn on. An additional message would be played to advise the mother to go to the toilet.
Prompt 2	Motion sensor	“*Mary, the toilet is upstairs*.”	

In accordance with the son's decision, it was agreed that IntraVox should verbalise a prompt directed to his mother (*Prompt 2*), and the prompt to Alexa (*Prompt 1*), to trigger home processes, would be implemented in future work. Therefore, whenever motion was detected in the food storage, Prompt 2 was played: “*Mary* [the mother's pseudo-name]*, the toilet is upstairs*.” Regarding the voice for the message, the son suggested that a familiar voice would be better than a synthesised one:

*Her dementia is very advanced, but she is strong-willed, and she will listen to a familiar voice, especially the carer's voice. She doesn't always listen to me, but she follows the carer's instructions really well*. (the son—family carer)

Hence, the prompt was verbalised by an occupational therapist who previously worked with the mother. As advised by the son, the prompt was repeated twice, and the system was installed in a place where it couldn't be reached or seen by his mother. This raised ethical issues about an intervention that was being done to a person rather than with a person, hence this issue was considered and balanced with other known priorities that the mother had long time expressed about her desire to be supported to live at home. Following deployment of IntraVox for 7 days, the son reported that there had been a positive change in his mother's behaviour:

*The idea is massive. The study was 100% successful as we did not have any accident in the food storage room since IntraVox was installed. The goal was achieved, she is not mistaking the utility room with the bathroom*. (the son—family carer)

The son mentioned he would recommend IntraVox to other people experiencing dementia symptoms and their family members and attributed the change in his mother's behaviour to the introduction of IntraVox in her home. Additionally, he felt that IntraVox could increase the quality of life of both for the carer and the cared for person:

*It can definitely improve her quality of life as sometimes she feels embarrassed of her actions. It's also amazing the amount of relief I have and this gives me more time to spend with my mother, rather than cleaning around the house*. (the son—family carer)

This indicates a positive change in a caring situation where it was becoming increasingly difficult for the son to provide care for his mother, and for this woman to increasingly experience the indignity of voiding in an inappropriate place. Previous research (Salai et al., 2021) indicates that taking the user's physical presence in the room into account should also be considered to avoid instances when IntraVox announces its action while the user is not present in the room. The resulting user experience of returning to a room to find certain aspects changed, e.g., the lights turned on, could be perceived as “*haunted*.” However, the son felt this was acceptable, as long as the situation was carefully monitored and any distress was immediately addressed by withdrawal of the technological intervention. He based his decision on his previous experience of technology installed in his mother's home:

*It's not scary, I already have all these smart devices that are doing things in the house. She would not be scared, she doesn't remember if the lights were on or off anyway*. (the son—family carer)

The son indicated that personalization of Prompt 2 (the voice, delivery pace and syntax) ensured that his mother could understand the message. He recognised that his mother's situation would change in time and this should be reflected in future use of this technological solution:

*We've put signs all over the house and she read them and took notice of them. In time, she started ignoring them. I'd say having two-three recordings in different voices would work, e.g., week 1—voice one, etc. then it becomes newer and she will listen to them. Definitely have two-three recordings of different family members, especially for early stages of dementia*. (the son—family carer)

## Discussion

The findings generated through the two studies reported in this paper provides support from family carers and professionals for the use of voice assistants (see also Astell et al., 2019; Sriram et al., 2019). This technology has the potential to provide reassurance and a sense of security for family carers, offer prompts, and reminders to the person with dementia to complete daily activities, provide alerts to enable carers to respond promptly, enhance home automation, and support family members to keep in touch. Our participants suggested that the use of the voice assistant was complementary to their caring role.

Yet there is also clear evidence that the use of a voice assistant was not without difficulties. Some family carers and professionals reported that the person they were supporting was unable to comprehend the purpose and function of a voice assistant, others were observed as unable to activate the technology, whilst others were more anxious when the synthetic voice of the assistant sounded without the presence of a person. Whilst these findings provide novel insight into the difficulties that carers experience when using a voice assistant, these findings also reflect the conclusions of Sriram, Jenkinson and Peters (2019) systematic literature review regarding informal carers' experience of assistive technology. This review draws attention to technology not meeting the needs of carers, and the requirement for the assistive technology to be adapted or customised. If ease of use and design flaws of technology are not addressed, carers can struggle and this can result in abandonment of the technology (Topo, 2008; Armstrong, 2012). For example, some functionality of voice assistants lacked interoperability with other technologies such as sensors to detect motion, touch, light, or sound. This could result in limited use of this technology, particularly in situations where the need is to support basic and Instrumental Activities of Daily Living for people living with dementia at home. Use of assistive technology for this purpose is somewhat neglected (Sriram et al., 2019), and should be further developed as this is a substantive aspect of a carer's role.

In the studies presented here there has been demonstration that design and usability problems could be overcome through technology, in this case IntraVox, that can create a virtual linking of the processing of sensors with the voice and home automation functionality of voice assistants. Moreover, this prototype could replace the synthetic voice of the assistant with a voice of a person known to the individual living with dementia. The pilot case study of IntraVox demonstrated how one woman recognised her carer's voice and responded appropriately by going to the bathroom rather than voiding in an inappropriate room in her home. Whilst IntraVox enabled processing of motion sensors and provided an instruction to go to the bathroom to urinate, the son carer anticipated the future requirement for additional input as behaviour changes may occur alongside advancing dementia. The full functionality of IntraVox processing sensor data and sending a programmed command to automate the turning on of the bathroom light could be a further technological solution. Whilst lighting interventions are non-invasive and have minimal adverse effects (Figueiro, 2017), this non-pharmacological intervention has largely been explored in the context of behavioural and psychological symptoms of dementia (Joa et al., 2022), rather than addressing Instrumental Activities, and this is a topic that should be considered in future research.

Previous research studies highlight the negative implications smart homes and assistive technologies might have on occupants with dementia (Wang et al., 2017; Tiersen et al., 2021). One consequence is that assistive technologies could decrease the interaction end-users have with their carers (Wang et al., 2017). The main purpose of Smart home technology in a caring context, in this case IntraVox, is to improve the quality of life of the end-user and reduce the burden professional carers and informal carers might sometimes experience. We therefore highlight that Smart home technology is not to be introduced in a home care context to avoid human contact and replace carers, but rather to address specific needs and problems that may benefit the person living with dementia.

Another issue study 2 raises is the inability to provide informed consent due to diminished capacity, leading to privacy concerns through use of Smart technology. Indeed, some results presented in this paper highlight the complex ethical dilemmas relating to voice assistants and use in caring contexts for people with dementia. For example, in reference to the individual who was unplugging their voice assistant, it's important to acknowledge that although the carer explicitly states that this unplugging is related to the person with dementia not recognising the technology or the person deeming it to be a fire risk, this could also be interpreted as a wish to stop using it. Whilst the technology was removed this does highlight an important point about learning an individual's communicative behaviours and monitoring their actions to ensure that their wishes and preferences are addressed (Hall et al., 2019). This also raises wider concerns about the need for the general public to understand the ethical considerations associated with the adoption of this technology for caring purposes and for further research into this topic. With regards to the IntraVox case study, we acknowledge that, given the mother's advanced state of dementia, she was not able to provide consent to take part in the study. Therefore, assent was obtained from an authorised consultee, i.e., her son (Slaughter et al., 2007). Personal consultees are charged with the responsibility to act in the best interests of another, in wanting to apply the least restrictive intervention aimed at enabling the cared for person to remain in their own home if that was their known aspiration. In the situation discussed in this paper decisions were based on the premise that best interests were served by attempting to use a technological solution to reduce a risk factor, voiding in an inappropriate place, that enhanced the likelihood of the breakdown of care leading to institutionalisation (Livingston et al., 2010).

Technology evaluation is essential in healthcare (Kjeldskov and Skov, 2014). Rogers et al. (2007) argue that laboratory studies are “*poor at capturing context of use*” and highlight that *in-situ* evaluations can indicate how people interact with technology in their intended setting. Klasnja et al. (2011) emphasise that small studies with considerable qualitative findings can address many design and usability issues before expanding to a large Randomised Control Trial. Bacchetti et al. (2011) highlight that studies of new technologies and ideas “*often must start small (sometimes even with n of 1) because of cost and feasibility concerns*.” Similarly, Caine (2016) notes that “*small*” sample sizes studies can reveal the most obvious usability problems and leave room to be replicated. On that line, Bradford and Zhang (2016) present the case of a participant taking part in a smart home pilot study that aimed to evaluate the efficacy of smart home sensors for tracking daily activities. Real-time data analysis was not implemented and unfortunately, the sensors did not trigger an alert one night regarding the participant's stroke that led to their death. When analysing the data leading up to the stroke, the authors noted that a combination of inferred activities, data derived from the sensors, and measurements from medical devices could have predicted that a stroke was imminent. Despite the small sample (*n* = 1), the case study brings important findings and demonstrates that smart home monitoring is effective in measuring daily activities and can also save the occupant's life.

Whilst the Intravox pilot case study was highly limited, and this does reduce transferability to other situations, it does provide a useful demonstration that interoperability could be achieved to enable exchanges between a range of products and voice assistants that provide customised and personalised technological solutions in a care context. Similar to Bradford and Zhang (2016), we believe that the preliminary findings obtained in this case study could inform future larger studies focusing on evaluating assistive technologies for prompting and supporting end-users with complex needs, such as dementia. However, it is important to highlight that such technological solutions should be carefully researched prior to further consideration as an intervention to support basic and instrumental *Activities of Daily Living* for people living with dementia at home. For example, although the familiar voice had a positive impact on the mother in the pilot case study, other users experiencing dementia symptoms might not want to hear the voice of a family member as it might be confusing for them (Salai et al., 2021). It is also possible that voice preferences could change in line with the severity of dementia and therefore, it is important to consider the needs and preferences of all end-users when designing such technologies for use in health and care contexts (Hall et al., 2019; Sriram et al., 2019).

In summary, the world is racing towards embracing voice assistants as part of a package of care to enable family carers and professionals to support people with cognitive problems to live at home. This is a point where there should be critical reflection and a need for co-production of this technology to ensure that it offers a personalised, adaptive technology that is fit-for-purpose in the context of care at home. There is a strong need for innovation and moving beyond simple sensors and robot-sounding voice automation to providing off-the-shelf products that can be customised without individuals or service providers having to invest heavily to make them actually usable. Only then will home care providers be able to offer these products as part of standard care and family carers have access to a wider range of products to support them in their caring role.

## Data availability statement

The datasets presented in this article are not readily available due to confidentiality, participants' privacy and in accordance with the ethical approval for the studies. Requests to access the datasets should be directed to glenda.cook@northumbria.ac.uk.

## Ethics statement

The studies involving human participants were reviewed and approved by Department of Nursing, Midwifery and Health, Northumbria University Ethics Committee. The patients/participants provided their written informed consent to participate in this study.

## Author contributions

GC conceived of the presented idea. GC and AK designed and analyzed the data for the first study. A-MS and LH designed and analyzed the data for the second study. All authors listed have made a substantial, direct, and intellectual contribution to the work and approved it for publication.

## Funding

We wish to thank Home Group and Sunderland City Council for financial support that enabled us to undertake these studies.

## Conflict of interest

The authors declare that the research was conducted in the absence of any commercial or financial relationships that could be construed as a potential conflict of interest.

## Publisher's note

All claims expressed in this article are solely those of the authors and do not necessarily represent those of their affiliated organizations, or those of the publisher, the editors and the reviewers. Any product that may be evaluated in this article, or claim that may be made by its manufacturer, is not guaranteed or endorsed by the publisher.
